# IL-10 Overexpression Reduces the Protective Response of an Experimental *Chlamydia abortus* Vaccine in a Murine Model

**DOI:** 10.3390/ani14162322

**Published:** 2024-08-12

**Authors:** Laura Del Río, Jesús Salinas, Nieves Ortega, Antonio J. Buendía, Jose A. Navarro, María Rosa Caro

**Affiliations:** 1 Departamento de Sanidad Animal, Facultad de Veterinaria, Regional Campus of International Excellence Campus Mare Nostrum, University of Murcia, 31000 Murcia, Spain; jsalinas@um.es (J.S.); nortega@um.es (N.O.); mrcaro@um.es (M.R.C.); 2 Departamento de Anatomía y Anatomía Patológica Comparadas, Facultad de Veterinaria, Regional Campus of International Excellence Campus Mare Nostrum, University of Murcia, 31000 Murcia, Spain; abuendia@um.es (A.J.B.); jnavarro@um.es (J.A.N.)

**Keywords:** IL-10, *C. abortus*, chronic infection, vaccine, mouse model, macIL-10 transgenic mice

## Abstract

**Simple Summary:**

*Chlamydia abortus* causes intracellular persistent infections in sheep, requiring a strong immune response for resolution. Interleukin-10 (IL-10) plays a dual role by modulating the inflammatory immune response during infection and preventing damage during pregnancy. This study used transgenic mice overexpressing IL-10 to model chronic *C. abortus* infections. The goal was to test the effectiveness of an experimental vaccine and understand the immune mechanisms involved when high levels of IL-10 are present. Both transgenic and control mice were vaccinated and exposed to the bacteria. Results showed that vaccinated control mice cleared the infection by day 9, while transgenic mice still had bacteria at day 28. Vaccination increased a key immune response, with IFN-*γ* and iNOS production, but did not change IL-10 levels in transgenic mice. Additionally, fewer immune cells were recruited in vaccinated transgenic mice. The study concludes that vaccine effectiveness is reduced in presence of high IL-10 levels. This model could be used for the development of better vaccines against *C. abortus*, and could help to test vaccines requiring strong cellular immune responses, thereby benefiting animal health.

**Abstract:**

In ovine populations, the enzootic nature of *Chlamydia abortus* (*C. abortus*) is attributed to its capacity to establish persistent intracellular infections, which necessitate a cellular immune response mediated by interferon-gamma (IFN-*γ*) for effective resolution. In both natural hosts and murine models, interleukin-10 (IL-10) has been demonstrated to modulate the cellular immune response crucial for the eradication of *C. abortus*. During gestation, it has also been shown to play a role in preventing inflammatory damage to gestational tissues and foetal loss through the downregulation of pro-inflammatory cytokines. This paradigm can be key for events leading to a protective response towards an infectious abortion. Previous research successfully established a mouse model of chronic *C. abortus* infection using transgenic mice overexpressing IL-10 (IL-10tg), simulating the dynamics of chronic infection observed in non-pregnant natural host. This study aims to evaluate the efficacy of an experimental inactivated vaccine against *C. abortus* and to elucidate the immune mechanisms involved in protection during chronic infection using this model. Transgenic and wild-type (WT) control mice were immunized and subsequently challenged with *C. abortus*. Vaccine effectiveness and immune response were assessed via immunohistochemistry and cytokine serum levels over a 28-day period. Morbidity, measured by daily weight loss, was more pronounced in non-vaccinated transgenic IL-10 mice, though no mortality was observed in any group. Vaccinated control mice eliminated the bacterial infection by day 9 post-infection (p.i.), whereas presence of bacteria was noted in vaccinated transgenic IL-10 mice until day 28 p.i. Vaccination induced an early post-infection increase in IFN-*γ* production, but did not alter IL-10 production in transgenic mice. Histological analysis indicated suboptimal recruitment of inflammatory cells in vaccinated transgenic IL-10 mice compared to WT controls. In summary, the findings suggest that IL-10 overexpression in transgenic mice diminishes the protective efficacy of vaccination, confirming that this model can be useful for validating the efficacy of vaccines against intracellular pathogens such as *C. abortus* that require robust cell-mediated immunity.

## 1. Introduction

*Chlamydia abortus* is an obligate intracellular bacterium that causes ovine enzootic abortion (OEA), markedly influencing livestock health and representing a zoonotic threat to pregnant women exposed to infected animals during the lambing period. A characteristic feature of OEA is its intracellular persistence. Sub-clinically infected hosts may sustain the propagation of this pathogen, thereby potentially undermining the efficacy of immunization protocols within the flock.

Previous studies utilizing an experimental intranasal infection model in pregnant sheep have indicated that pregnancy outcomes may hinge on the infectious dose, with a robust antibody response correlating with protection [1]. It has also been shown that IFN-*γ* and IL-10 are produced by peripheral blood mononuclear cells (PBMC) during both the intracellular persistent stage following infection and the active disease phase in both protected and aborted sheep [2]. In mice, pathogenesis of abortion mimics the events shown in the natural host; foetal loss has been related to bacterial colonization in the placenta [3] and to the expression of pro-inflammatory cytokines such as IFN-*γ* and TNF-*α* in the last third of gestation [4]. However, differences in placental cytokine expression between mice and sheep have been described [5]. Currently, there is a need to further characterize the immunological mechanisms that underlie the dormancy and subsequent reactivation of bacteria during the last stages of gestation, and more importantly, the events that underlie full protection.

Given the challenges inherent in employing natural host models, an in vivo murine model that replicates chlamydial sub-clinical/chronic infection could prove invaluable.

Indeed, murine models have been extensively employed within chlamydial research to elucidate host immune mechanisms after primary infection and during pregnancy as well as to advance vaccine development. However, chlamydial persistence in such in vivo models is typically transient within the tissues (reviewed in [4]). Specifically, infection in resistant mouse strains elicits a potent inflammatory response, facilitating rapid bacterial clearance from tissues, whereas susceptible strains experience systemic infection, leading to immunopathogenesis and mortality. Consequently, in order to thoroughly investigate the immunological processes during chlamydial intracellular persistence and bacterial reactivation, a sustained *C. abortus* infection model was established utilizing transgenic mice overproducing IL-10 [6]. It is important to note that while macrophages in IL-10-tg mice exhibit elevated IL-10 expression, they are reported to remain fully immunocompetent, mounting an appropriate immune response post-infection [7].

In both natural and experimental murine hosts, *C. abortus* infection provokes the secretion of Th1 cytokines such as interferon (IFN-*γ*) and tumor necrosis factor TNF-*α*, which is pivotal in orchestrating a protective cellular response against the pathogen (reviewed in [4,5]).

IL-10 is critically involved in immune defense against *Chlamydia* by moderating inflammation and antigen presentation. In fact, the absence of IL-10 has been shown to be associated with the prevention of intracellular inflammasome assembly and dendritic cell apoptosis, allowing for more efficient antigen presentation and consequently an intensified Th1 cell immune response, which is essential for counteracting chlamydial infections in IL-10 knockout mice [8,9]. IFN-*γ* is considered a crucial component of the protective type 1 response to *Chlamydia*; as an inhibitory cytokine, IL-10 can diminish the expression of Th1 cytokines, including IFN-*γ*, thereby enabling intracellular bacterial persistence and chronic infection.

Understanding the role of IL-10 in vaccine efficacy is critical. It has been documented that endogenously produced IL-10 plays a significant role in the establishment and maintenance of infections by intracellular pathogens, including viruses [10]. Moreover, research into the impact of IL-10 on immune responses has revealed that IL-10 deficiency promotes enhanced antigen-specific Th1 responses following vaccination [11], highlighting the potential of targeting IL-10 modulation to bolster vaccine-induced protection against intracellular pathogens.

However, the function of IL-10 in the persistence of chlamydial infections remains inadequately understood, and the possibility of therapeutically inhibiting IL-10 to amplify anti-chlamydial immunity and improve vaccine effectiveness has yet to be explored in the context of animal chlamydiosis.

This study seeks to assess the effectiveness of an experimental inactivated vaccine targeting *C. abortus* in the context of chronic infection utilizing this murine model and to delineate the immunological mechanisms contributing to protection.

## 2. Materials and Methods

### 2.1. Ethics Statement

Female mice of FVB background (control WT mice) and transgenic mice overexpressing IL-10 (IL-10-tg) were kept in a specific pathogen-free facility at the University of Murcia. Handling and experimental procedures were design and carried out to minimize animal suffering in accordance with the principles of the Basel Declaration and Spanish legislation concerning the protection of the animals for experimentation (RD 53/2013). This study protocol was approved by the Committee on the Ethics of Research in Animal Experimentation of the University of Murcia, Spain (Protocol number: C1310050301).

### 2.2. Microorganisms

The AB7 strain of *C. abortus* was propagated in the yolk sacs of developing chick embryos and the chlamydial titers were determined by counting inclusion-forming units (IFUs) in McCoy cells, following the methodology described by Buendia et al. (1999) [12]. Aliquots of the bacteria were standardized and stored at −80 °C until required for use.

### 2.3. Animals, Vaccination, and Experimental Design

Female control mice of FVB background (wild type, WT) and IL-10 overexpressing transgenic (IL-10-tg) mice eight weeks of age received two doses of an experimental inactivated vaccine, formulated as described in [13] and previously tested and validated in our laboratory [13,14,15,16]. Vaccinated mice received two doses of the vaccine: 15 μg of *C. abortus* proteins in 0.2 mL subcutaneously 44 and 30 days before the infection, using a purified derivate of saponin (QS21, Agenus) as adjuvant.

**Figure 1:**
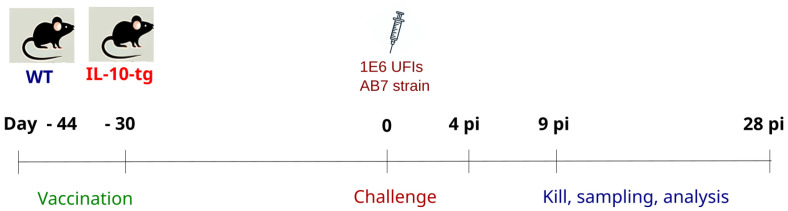
Vaccination and experimental infection. WT and IL-10-tg vaccinated mice received two doses of vaccine (15 μg of *C. abortus* proteins in 0.2 mL) subcutaneously 44 and 30 days before the infection. Mice were intraperitoneally challenged with 10^6^ inclusion-forming units (IFUs) of *Chlamydia abortus* in 0.2 mL of 0.1M phosphate-buffered saline (PBS), pH 7.2, and monitored daily. Mice were euthanized at 4, 9, and 28 days post-inoculation).

Mice were challenged with 10^6^ inclusion-forming units (IFUs) of *Chlamydia abortus* in 0.2 mL of 0.1 M phosphate-buffered saline (PBS), pH 7.2 intraperitoneally and monitored daily. Non-vaccinated challenged control groups (WT-c and IL10-tg-c) consisted of 5–7 mice each and received 0.2 mL of sterile PBS. These mice were euthanized at 4, 9, and 28 days post-inoculation (dpi.) (Figure 1). During necropsy, blood samples were obtained via cardiac puncture and liver tissue samples were preserved in either 10% formalin or a Zinc fixative agent (BD Biosciences, Pharmingen, San Jose, CA, USA), then embedded in paraffin for histopathological or immunopathological evaluations. All experimental procedures were conducted in duplicate.

### 2.4. Morbidity and Course of Infection

The mice were observed daily for signs of infection and morbidity was assessed based on the average daily weight loss per group. In addition, the effect of the vaccine on the progression of *C. abortus* infection in mice was compared through the bacterial clearance in liver tissue. The level of infection was quantified by titrating the inclusion-forming units (IFUs) per gram of tissue on McCoy cell monolayers, as previously detailed in [12].

### 2.5. Histopathology and Immunohistochemistry

Formalin-fixed liver sections (4 μm) were stained with haematoxylin–eosin staining for histopathological study, and immunohistochemical labeling was carried out to demonstrate presence of chlamydial antigen (Abcam, Cambridge, UK) as previously described [12]. Additionally, characterization of the inflammatory infiltrate was carried out in Zinc-fixed samples using monoclonal antibodies against iNOS, as described in [17]. Isotype control antibodies were used to assess the nonspecific background staining, as described in [15].

### 2.6. Cytokine Levels in Serum

In addition, levels of cytokines in the serum were measured using commercial kits. Briefly, samples were collected from mice at the time of killing and analyzed for detection IFN-*γ* and IL-10 cytokines by ELISA commercial kits (R & D Systems Inc., Minneapolis, MN, USA) following the manufacturer’s instructions.

### 2.7. Statistical Analysis

To compare the means of two independent samples (WT and IL-10-tg groups or control and vaccinated groups), a non-parametric Mann–Whitney U test was used. A one-way ANOVA test was used to compare means between WT and IL-10-tg groups and changes in weight loss or bacterial level over time, with number of IFUs as the measurement variable and mouse strain, time after infection, and vaccination as the factors. As a post hoc test, Tukey’s multiple comparisons of means with 95% confidence level was used. All analyses were performed using R (R version 3.4.4) [18], and criteria for reproducible research were followed [19]. A *p* value of <0.05 was considered statistically significant.

## 3. Results

### 3.1. Morbidity and Course of Infection

**Figure 2:**
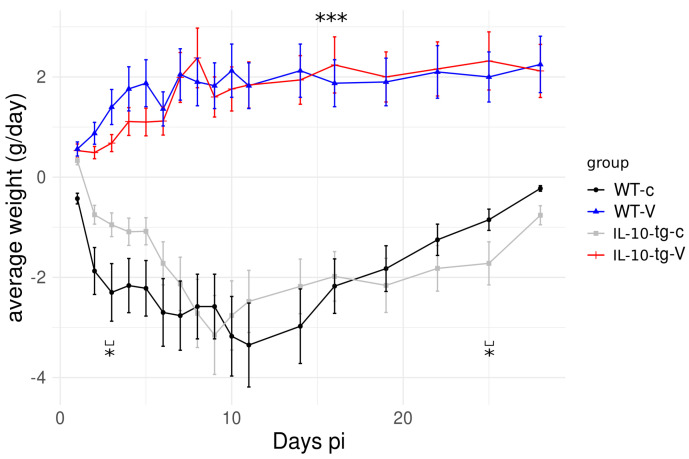
Morbidity, measured as daily weight loss, in IL-10-tg and wild-type mice (WT). Weight loss was only significant in vaccinated (V) as compared to control non-vaccinated (c) groups. Weight loss and slower recovery was observed in IL-10-tg-c mice 20 days p.i. No mortality was observed in any of the groups. (*** *p* < 0.001; * *p* < 0.05).

Morbidity, measured as weight loss, was monitored daily in IL-10-tg and WT mice, both vaccinated (V) and non-vaccinated (c). No weight loss was observed in (V) animals, while both mouse strains showed minimal differences in weight loss following vaccination; however, slower recovery was shown by the transgenic mice, with significant differences noted only on day 24 p.i. (Figure 2). Mortality was not observed in any of the groups.

### 3.2. Bacterial Isolation

**Figure 3:**
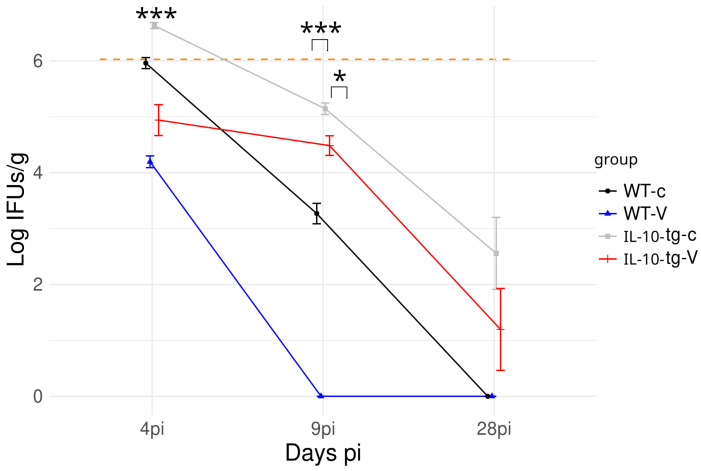
Temporal analysis of *C. abortus* infection levels in liver tissue after infection as measured by IFUs/g. Although a reduction in infection levels was noted in all mouse groups, only the vaccinated WT group (WT-V) was able to clear the bacteria from the liver by day 9 p.i. At 28 days p.i., *C. abortus* was detected only in IL-10-tg mice, which was the case for both control (tg-c) and vaccinated (tg-V) groups. The orange line marks the challenge dose at day 0 p.i. (*** *p* < 0.001; * *p* < 0.05).

Regarding the course of infection, *C. abortus* isolation was carried out from liver samples. The liver is recognized as a target organ following intraperitoneal infection, exhibiting infection kinetics similar to those observed in the spleen [20]. A high presence of *C. abortus* at 4 and 9 dpi. was observed in all groups excepting the WT vaccinated mice group, where the presence of *C. abortus* was not detected from 9 dpi. (Figure 3). At 28 dpi., *C. abortus* was detected only in both IL-10-tg groups, although in lower levels than at 4 and 9 dpi.

### 3.3. Histopathological and Immunohistochemical Findings

**Figure 4:**
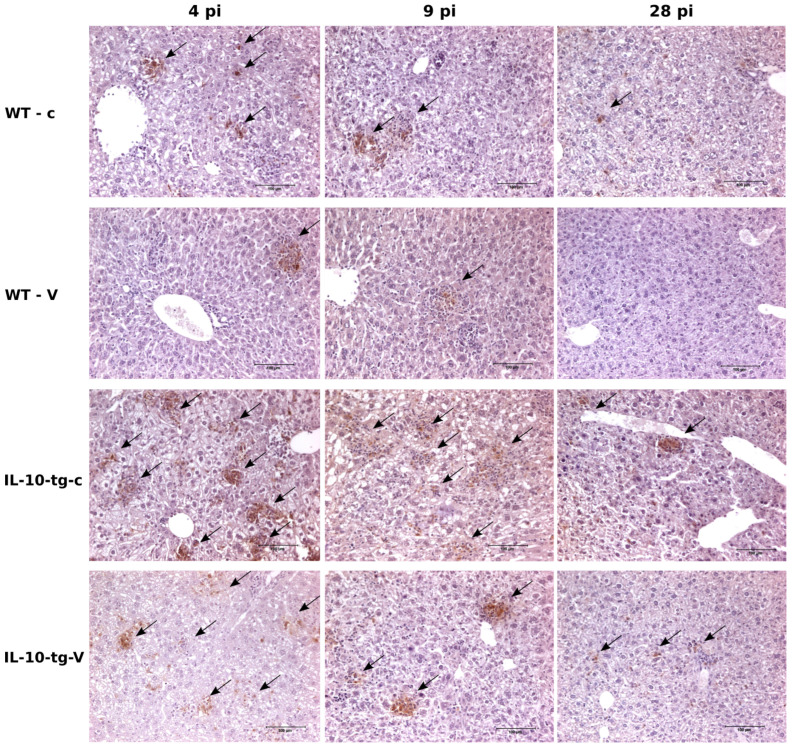
Inflammatory response and chlamydial antigen levels in WT and IL-10-tg mice at different time points after infection. At day 4 p.i., both WT and IL-10-tg mice developed multifocal hepatitis with high levels of chlamydial antigen, even after vaccination. WT vaccinated (WT-V) mice exhibited inflammation predominantly with macrophages and PMNs, while IL-10-tg (IL-10-tg-V) mice had fewer and smaller foci dominated by macrophages. By day 9 p.i., lymphocytes predominated at inflammatory sites in both strains. By day 28 p.i., the presence of small inflammatory foci with chlamydial antigen in IL-10-tg-V suggested a reduced protective effect of vaccination compared to WT-V mice. Black arrows indicate the presence of chlamydial antigen in the tissue sections.

Multifocal granulomatous hepatitis associated with presence of chlamydial antigen was detected in transgenic vaccinated mice (IL-10-tg-V), transgenic non-vaccinated mice (IL-10-tg-c), and wild-type non-vaccinated mice (WT-c) at days 4, 9, and 28 dpi., but was not detected in wild-type vaccinated mice (WT-V) at 28 p.i. (Figure 4). The characterization of inflammatory infiltrate was composed by macrophages and variable amounts of PMN or T cells (PMN mainly at day 4 p.i. and T cells at day 9 p.i). The higher amount of chlamydial antigen at day 4 p.i. was observed in IL-10-tg-c mice, while the number and size of inflammatory foci was lower than in WT mice. At day 9 p.i., a decrease of chlamydial antigen was observed in all groups except IL-10-tg mice, where a multifocal hepatitis and chlamydial antigen was observed. At day 28 p.i., neither antigen nor inflammation was observed in WT-V mice; some chlamydial antigen and hepatitis could be observed in the other groups of mice, with a higher level in IL-10-tg-c mice particularly.

### 3.4. Immune Response

**Figure 5:**
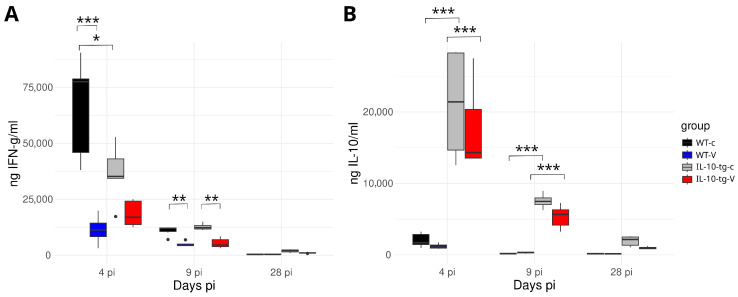
IFN-*γ* and IL-10 levels in sera at different time points after infection: (**A**) IFN-*γ* had significantly higher values in the non-vaccinated groups, particularly in WT mice; (**B**) IL-10 was significantly elevated in IL-10-tg overexpressing mice as compared to WT mice at 4 and 9 days p.i. in both the control and vaccinated groups (tg-c and tg-V). (Black dots represent individual data points beyond the interquartile range. *** *p* < 0.001; ** *p* < 0.01; * *p* < 0.05).

High levels of IFN-*γ* were detected at day 4 p.i., with vaccination significantly reducing these levels in both WT and IL-10-tg mice (WT-V and IL-10-tg-V) (Figure 5A). Conversely, IL-10 levels were consistently higher in IL-10-tg mice than in WT mice for both control and vaccinated groups (IL-10-tg-c and IL-10-tg-V); see Figure 5B.

**Figure 6:**
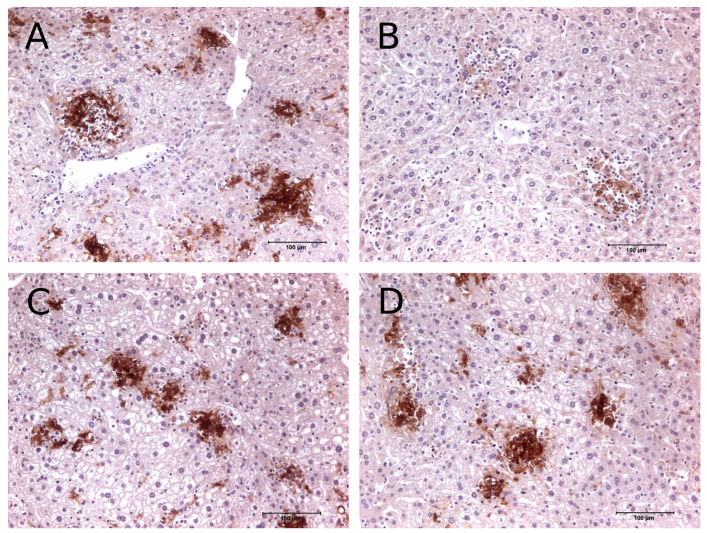
Expression of iNOS at day 4 p.i. in the liver. While iNOS expression remained unaffected in IL-10-tg mice, it was significantly reduced in the WT control group following vaccination ((**A**) WT non-vaccinated; (**B**) WT vaccinated). In contrast, the expression of iNOS was strong in the liver macrophages of IL-10-tg mice ((**C**) IL-10-tg non-vaccinated; (**D**) IL-10-tg vaccinated).

The expression of iNOS was not depleted in the liver macrophages of transgenic mice after vaccination (IL-10-tg-V), with a pattern similar to non-vaccinated mice at 4 dpi. In contrast, wild-type vaccinated mice (WT-V) showed clearly weaker expression (Figure 6).

## 4. Discussion

Our study demonstrates the complex interplay between IL-10 production and the protective response to an experimental *C. abortus* vaccine in transgenic mice. The overexpression of IL-10 facilitated the establishment of a long-term infection by the microorganism in the liver tissue, similar to findings in a previous study [15], where non-vaccinated mice exhibited impaired infection clearance due to decreased macrophage recruitment, leading to more diffuse inflammation. Furthermore, although both mouse strains showed similar improvements in symptoms following vaccination, with minimal differences in weight loss recovery, significant differences were observed on day 24 p.i. between the transgenic and wt mice, with the former showing slower recovery. This observation aligns with prior research indicating that elevated IL-10 levels can impair the immune system’s ability to control chlamydial infections effectively [7].

Moreover, the immunosuppressive effect of IL-10 in regulating immune responses during infection with intracellular pathogens is well documented [21]. Specifically, in other members of the Chlamydia family, IL-10 knockout mice infected with these pathogens showed faster clearance in the absence of IL-10, albeit with increased immunopathology and inflammation in the tissue [22]. Additionally, in *Brucella abortus* infections, the persistence of this microorganism has been linked to IL-10 production by regulatory CD4+CD25+ T cells [23]. In addition, other models of sustained tissue infection have been described for *Coxiella burnetti* using overexpressing transgenic mice, where chronic infection was associated with impaired bactericidal pathways in macrophages [24].

Similarly, in our study, a delay in clearing bacteria from day 9 p.i. onward was observed in vaccinated IL-10 tg mice compared to WT mice. This bacterial clearance was likely due to an effective immune response facilitated by vaccination. In addition, immunohistochemical analysis showed multi-focal granulomatous hepatitis associated with chlamydial antigen and IL-10 overproduction, indicating compromised ability to control early infection stages. This finding supports previous studies in which IL-10 overexpression hindered bacterial clearance, leading to prolonged infection [6], suggesting an impaired protective response due to the immunosuppressive effects of IL-10, as has been previously described [8]. In IL-10-tg mice, the prolonged presence of inflammatory foci and immune reactions in the liver post-infection, even in vaccinated subjects, may be attributed to a delay in chlamydia clearance, potentially below the detection threshold of current isolation techniques. Additionally, the monoclonal antibody we used is reactive to any antigen present in the tissue, which may indicate the presence of non-infectious forms of chlamydia (such as reticulate bodies, aberrant forms, or phagocytosed remnants of chlamydial membranes) or a combination of both factors.

Additionally, our study revealed that vaccination significantly reduced IFN-*γ* levels in both WT and IL-10-tg mice. This reduction in IFN-*γ* levels following vaccination suggests effective modulation of the immune response, promoting a more balanced cytokine profile conducive to bacterial clearance without triggering over-inflammation. However, IL-10 levels remained consistently higher in IL-10-tg mice irrespective of vaccination, indicating that IL-10 overexpression sustains an environment favoring persistent infection, as previously shown during *Coxiella burnetti* infection [24]. One limitation of this study is the lack of pre-challenge measurements for IFN-*γ* and IL-10 levels. Future studies should consider incorporating these baseline measurements in order to better understand the impact of vaccination on cytokine levels and subsequent challenge responses. Previous findings have shown that IL-10’s anti-inflammatory mechanisms include the suppression of both macrophage and dendritic cell antigen-presenting functions and the inhibition of pro-inflammatory cytokines [25].

Furthermore, we have described the higher expression of iNOS in liver macrophages in IL-10-tg vaccinated mice. This finding indicates that vaccination triggers a different expression of this microbicidal pathway compared to primary infection [15]. This elevated iNOS production in IL-10-tg vaccinated mice could be associated with the presence of viable bacteria within cells and the inflammatory response in the tissue. Although experimental studies using iNOS-deficient mice have determined that production of NO by iNOS is needed for protective immune response to a wide variety of microbial infections (reviewed in [26]), it has been found that the production of NO in *M. tuberculosis* infection can limit tissue damage that would otherwise result from a persistently active innate immune response [27]. Therefore, the role of iNOS during intracellular infections has been shown to be complex, and its effects can be influenced by both the microorganism and the stage of infection [26].

In summary, our results show that IL-10 overexpression decreased the efficacy of an experimental inactivated vaccine that has been proven to induce a good protective immune response against *C. abortus* in both mice and the natural ovine host [4,13,14,15,16]. Our findings highlight the critical role of IL-10 in modulating immune responses to *C. abortus* infection and the opportunities that it poses for vaccine efficacy, as shown by other studies of *C. trachomatis* infection [9].

Interestingly, other studies using the BCG vaccine have shown that interfering with IL-10 signaling can result in long-term protection after vaccination by altering memory T cell populations [28]. As a result, targeting IL-10 pathways could be considered as a way to enhance vaccine effectiveness, potentially leading to better control of chlamydial infections, as has been suggested by other authors [21,25].

This IL-10 tg mouse model could be a valid way to evaluate the efficacy of vaccines against OEA and other intracellular pathogens for which a strong cell-mediated immune response is required to protect against chronic or persistent infections [13].

## 5. Conclusions

1. IL-10 overexpression reduces vaccine efficacy in transgenic mice, accompanied by higher levels of infection in the liver and iNOS expression.
2. The IL-10-tg mouse strain may serve as a suitable and robust model for future evaluations of vaccine effectiveness against chronic infections of *C. abortus*.

## Data Availability

Data are contained within the article.

## Abbreviations

The following abbreviations are used in this manuscript:

ELISA	Enzyme-Linked Immunosorbent Assay
IFN-*γ*	Interferon-*γ*
IFUs	Inclusion-Forming Units
IL-10	Interleukin-10
iNOS	Inducible Nitric Oxide Synthase
OEA	Ovine Enzootic Abortion
PBMC	Peripheral Blood Mononuclear Cells
PMNs	Polymorphonuclear Neutrophils
